# The association between health literacy and e-cigarette use: evidence from Zhejiang, China

**DOI:** 10.3389/fpubh.2023.1321457

**Published:** 2024-01-04

**Authors:** Xuehai Zhang, Xinxin Zhang, Songjia Zhang, Lizheng Ge, Yue Xu, Dingning Yao, Xiujing Hu, Zishuo Huang, Tingting Zhu, Zixia Wang, Chun Chen

**Affiliations:** ^1^Zhejiang Provincial Center for Disease Control and Prevention, Hangzhou, China; ^2^School of Public Health and Management, Wenzhou Medical University, Wenzhou, China; ^3^School of Innovation and Entrepreneurship, Wenzhou Medical University, Wenzhou, China; ^4^Sir Run Run Shaw Hospital, Affiliated with the Zhejiang University School of Medicine, Hangzhou, China; ^5^School of Business, Macau University of Science and Technology, Macau, China

**Keywords:** health literacy, electronic cigarettes, conventional cigarettes, dual use, multinomial logistic regression, 2022 China health literacy survey

## Abstract

**Objectives:**

The worldwide popularity of electronic cigarettes (ECIG) is becoming a public health concern. Compared to conventional cigarettes (CIG), the harm caused by ECIG is more insidious. Studies have shown that lower health literacy (HL) is associated with CIG use; however, the relationship between HL and ECIG use remains controversial. Because ECIG emerged more recently than CIG, there are fewer relevant studies, and the sample populations and evaluation methods of HL in existing studies differ. This study conducted a large-sample survey to examine the relationship between HL and ECIG use.

**Methods:**

As part of the 2022 China Health Literacy Survey, a total of 60,998 valid questionnaires were collected from September to November in 2022 using a stratified multistage probability proportional to the population size sampling frame. Chi-square tests and multinomial logistic regression was used to analyze the relationship between HL and ECIG use. Some demographic variables were included as covariates in the analysis.

**Results:**

The study showed that the average HL score and the HL level of Zhejiang residents in 2022 were 42.8 and 30.8%, respectively. The prevalence of CIG and ECIG was 19.7 and 1.0%, respectively; 19% of participants exclusively used CIG, while only 0.3% of participants used ECIG exclusively; dual users accounted for 0.6%. After adjusting for covariates, adequate HL was associated with lower odds of ECIG-exclusive use (odds ratio [OR] = 0.452, *p* < 0.001), CIG-exclusive use (OR = 0.833, *p* < 0.001), and dual use (OR = 0.632, *p* < 0.001). Young age, male sex, unmarried status, high-income status, and absence of chronic disease were also associated with ECIG use.

**Conclusion:**

HL was a protective factor against both patterns of ECIG use, especially ECIG-exclusive use. Health policymakers and public health practitioners should consider HL as a potential measure for ECIG control.

## Introduction

Conventional cigarette (CIG) use has been proven to be associated with negative health conditions, such as cardiovascular diseases and lung cancer (1–3). Consequently, laws have been introduced worldwide to restrict CIG use (4, 5), and a variety of smoking cessation products have emerged. Since 2007, electronic cigarettes (ECIG) have been swiftly promoted (6–8) as an alternative to CIG. People believe that ECIG use is less harmful and addictive; however, in practice, its use has been proven hazardous to human health (9, 10). The US Centers for Disease Control and Prevention recommend young adults not use ECIG or vaping products (containing nicotine or tetrahydrocannabinol) because of the effects of nicotine on brain development (11). ECIG use among Chinese adults aged 18–29 increased significantly from 2.0% in 2015–16 to 2.7% in 2018–19 (11). China currently regulates the sale and promotion of ECIG only for those under the age of 18 years (12, 13). In addition to legislation, research on the factors influencing ECIG use can provide policymakers with additional ECIG control ideas.

Health literacy (HL) refers to the ability of individuals to access and understand health information and use this information to maintain and promote their own health (14). Existing literature suggests that HL contributes to the adoption of healthy behaviors (15, 16), including reduced smoking behavior (17, 18). Recent studies have shown that individuals with low HL have higher rates of CIG use compared to those with high HL (19). In addition, there is literature suggesting that different levels of HL were associated with prevention of smoking (20).Thus, improving HL is a potential measure for controlling smoking behavior. Does HL have the same effect on ECIG use? Few studies have examined this question, and the results are controversial (21–23). Therefore, we conducted a large-sample survey of 60,998 people in Zhejiang Province, China, to address the role of HL in ECIG use.

Relevant surveys have shown that most ECIG users are dual users and have converted from being CIG-exclusive users (11). Therefore, ECIG users comprise two groups: dual users and ECIG-exclusive users. The HL status of these two groups likely differs. To eliminate potential confusion, we divided tobacco users into three categories: CIG-exclusive, ECIG-exclusive, and dual users, and then calculated the odds ratio (OR) of each group separately.

With its increasing popularity, ECIG is becoming the first tobacco product to which young people are exposed. It is important to clarify the relationship between HL and ECIG use. We wanted to determine whether HL remains significantly associated with ECIG use after excluding the effects of CIG use. We then provide a reference for policymakers regarding ECIG control.

## Methods

### Study design

This cross-sectional study was conducted in Zhejiang Province as part of the 2022 China Health Literacy Survey. The target population of this survey is the non-collective resident population with Chinese nationality aged 15–69 years old in all counties (cities and districts) in Zhejiang Province, excluding residents living in military bases, hospitals, prisons, nursing homes, and dormitories. The permanent population comprises residents who have resided in the local area for more than six months in the past 12 months, regardless of whether they have a local household registration status. We excluded people under the age of 15 years because this is the age at which compulsory education has typically not been completed. We also excluded people over the age of 69 years, because people over this age are more likely to have cognitive impairment (24).

The survey was voluntary and anonymous, and this information was communicated to participants prior to the survey. Potential participants were asked to indicate that they had read and understood the consent instructions information and agreed to participate before taking the questionnaire. Questionnaires were completed through face-to-face interviews, and the interviewers had received professional training in advance. This study was reviewed and approved by the Research Ethics Committee of Zhejiang Provincial Center for Disease Control and Prevention.

### Sampling methods

The minimum sample size for each county (city or district) is calculated as N = $\frac{\mu_{\alpha }^{2}\times p\times \left(1-p\right)}{\delta^{2}}$ ×*deff*. According to the HL level of Zhejiang Province in 2021—36.11%—p is set as 0.3611; the allowable relative error is 15%; the allowable absolute error δ = 0.3611 × 0.15 = 0.0542, μ_α_ = 1.96, deff = 1, and the minimum sample size per county is finally calculated as 302. Considering the invalid questionnaires and the low vaping rate of e-cigarettes, we expanded the sample size to 640 per county (city or district).

This survey covered all 90 counties (cities or districts) in Zhejiang Province. The field investigation officially began in September 2022 and was completed by the end of November 2022. Sampling methods were carried out in accordance with national guidance (25), which was divided into the following four steps: (1) each county (city, district) adopted stratified multistage probabilities proportional to the population size (PPS) sampling frame to select four townships; (2) each township adopted a PPS sampling frame to select two communities (villages); (3) 100 households were selected from each community (village) by random sampling; and (4) one participant from each household was selected for a face-to-face interview using Kish’s grid (26). At least 80 participants were required to complete the questionnaire for each community (village). Through the above sampling steps, we collected a total of 60,998 qualified questionnaires.

### Measures

We adopted the Zhejiang Citizen Health Literacy and Tobacco Use Questionnaire (2022), which was modified from the Chinese Citizen Health Literacy Questionnaire (2022). The questionnaire comprised three parts (HL, Tobacco Use, and Personal Characteristics) and was issued by the Zhejiang Provincial Center for Disease Control and Prevention.

The Chinese Health Literacy Scale was used to assess HL in the first part of the questionnaire. This scale was developed by the Chinese Center for Health Education using the Delphi method (27). The scale consists of 50 items and is divided into three dimensions: knowledge and attitudes, behavior and lifestyle, and health-related skills (28). The scale comprises three types of questions: true or false (one point for a correct answer), single answer (choose the only correct answer from several available answers and score one point for a correct response) and multiple answers (several possible answers are correct; a “correct response” comprises selecting all the correct answers and two points are awarded for a correct response). A score of 53 out of 66 (80%) is considered to have adequate HL, and a score of 0–52 is considered to have limited HL. The overall Cronbach’s alpha for the scale was 0.95, and the Spearman-Brown coefficient was 0.94 (29).

The second part contained seven questions related to tobacco use; we analyzed two of them, “Do you currently smoke conventional cigarettes?” and “Do you currently use e-cigarettes?” These two questions have four possible answers: 1 “yes, I do, and I smoke every day”; 2 “yes, I do, but not every day”; 3 “I used to smoke, but have now quit”; and 4 “I have never smoked.” Participants were classified as users of a particular tobacco product if they chose answers 1 or 2 and as non-users if they chose answers 3 or 4. A 4-level categorical variable was calculated. Those who chose 3 or 4 for both questions were defined as non-users and coded as 1. Using only CIG and not ECIG was defined as a CIG-exclusive user and coded as 2. Using only ECIG and not CIG denoted an ECIG-exclusive user and was coded as 3. Dual users were defined as those who use both CIG and ECIG and were coded as 4.

At the end of the questionnaire, we collected personal characteristics, some of which were used as covariates in logistic regression. Sex was included as a covariate as a binary variable (male/female) because studies have reported that males are much more likely to use tobacco than females (30). Age was regarded as a four-level ordinal variable (15–24, 25–44, 45–64, 65–69 years); studies have found that middle-aged (25–64 years) people are associated with more tobacco use than other age groups (31). Marital status is divided into married and other status (single/widow/divorced) because studies have reported that married people use less tobacco than other married status groups (32). Educational level was considered a three-level ordinal variable (less than junior high school, junior/senior high school, and college or above); previous studies have suggested that people with less education tend to use tobacco more frequently (33). Occupation was treated as a five-level categorical variable (technical/professional, commercial/service, student, manual, unemployed/other). Previous studies have demonstrated that managers and/or professionals are more likely to smoke (33). Annual household income was treated as a four-level ordinal variable (0–49,999, 50,000–99,999, 100,000–149,999, or ≥ 150,000 Yuan). Previous research has shown that smoking is more widespread among the least well-off (34). Chronic condition was a binary variable (yes/no) because many studies have reported associations between smoking and chronic diseases (35).

### Statistical analysis

Data were processed in SPSS Statistics, version 25.0. The significance level was set as *p* < 0.05. First, we conducted a descriptive analysis of the sample data, showing the frequency and percentage of all variables (Table 1). Chi-square tests were then used to examine whether each variable was associated with tobacco use patterns (Table 1). Finally, multinomial logistic regression was used to test whether HL independently affected ECIG use (Table 2). In the multinomial logistic regression model, the outcome reference group was tobacco non-users.

**Table 1:** Composition of tobacco use by participant characteristics.

Variable		N (%)	Non-user	CIG-exclusive	ECIG-exclusive	Dual-use	χ^2^ (value of p)
	Total	60,998(100.0)	48,812 (80.0)	11,594 (19.0)	196 (0.3)	396 (0.6)	
Health literacy	Adequate	18,764 (30.8)	15,584 (83.1)	3,014 (16.1)	44 (0.2)	122 (0.7)	**161.078** **(p < 0.001)**
	Limited	42,234 (69.2)	33,228 (78.7)	8,580 (20.3)	152 (0.4)	274 (0.6)	
Sex	Male	28,987 (47.5)	16,985 (58.6)	11,492 (39.6)	124 (0.4)	386 (1.3)	**15962.638** **(p < 0.001)**
	Female	32,011 (52.5)	31,827 (99.4)	102 (0.3)	72 (0.2)	10 (0.1)	
Age	15–24	2,521 (4.1)	2,376 (94.2)	110 (4.4)	7 (0.3)	28 (1.1)	**602.123** **(p < 0.001)**
	25–44	17,902 (29.3)	14,697 (82.1)	2,957 (16.5)	81 (0.5)	167 (0.9)	
	45–64	32,125 (52.7)	24,981 (77.8)	6,884 (21.4)	84 (0.3)	176 (0.5)	
	≥65	8,450 (13.9)	6,758 (80.0)	1,643 (19.4)	24 (0.3)	25 (0.3)	
Marital status	Single/Widow/Divorced	10,903 (17.9)	8,698 (79.8)	2045 (18.8)	50 (0.5)	110 (1.0)	**34.783** **(p < 0.001)**
	Married	50,095 (82.1)	40,114 (80.1)	9,549 (19.1)	146 (0.3)	286 (0.6)	
Educational level	Less than junior high school	17,809 (29.2)	14,545 (81.7)	3,141 (17.6)	48 (0.3)	75 (0.4)	**676.157** **(p < 0.001)**
	Junior/senior high school	29,970 (49.1)	22,874 (76.3)	6,798 (22.7)	100 (0.3)	198 (0.7)	
	College or above	13,219 (21.7)	11,393 (86.2)	1,655 (12.5)	48 (0.4)	123 (0.9)	
Occupation	Technical/Professional	7,179 (11.8)	6,078 (84.7)	1,019 (14.2)	33 (0.5)	49 (0.7)	**533.947** **(p < 0.001)**
	Students	1,339 (2.2)	1,310 (97.8)	21 (1.6)	3 (0.2)	5 (0.4)	
	Manual	31,638 (51.9)	24,593 (77.7)	6,775 (21.4)	90 (0.3)	180 (0.6)	
	Commercial/Service	11,902 (19.5)	9,574 (80.4)	2,168 (18.2)	46 (0.4)	114 (1.0)	
	Unemployed/Others	8,940 (14.7)	7,257 (81.2)	1,611 (18.0)	24 (0.3)	48 (0.5)	
Annual household income	0–49,999	18,814 (30.8)	15,056 (80.0)	3,565 (18.9)	69 (0.4)	124 (0.7)	**40.365** **(p < 0.001)**
	50,000–99,999	14,282 (23.4)	11,344 (79.4)	2,822 (19.8)	43 (0.3)	73 (0.5)	
	100,000–149,999	12,944 (21.2)	10,312 (79.7)	2,531 (19.6)	32 (0.2)	69 (0.5)	
	≥15,0000	14,958 (24.5)	12,100 (80.9)	2,676 (17.9)	52 (0.3)	130 (0.9)	
Chronic conditions	No	44,049 (72.2)	35,468 (80.5)	8,122 (18.4)	146 (0.3)	313 (0.7)	**41.566** **(p < 0.001)**
	Yes	16,949 (27.8)	13,344 (78.7)	3,472 (20.5)	50 (0.3)	83 (0.5)	

Boldface indicates statistical significance (*p < 0.05; **p < 0.01; ***p < 0.001).

**Table 2:** Multinomial logistic regression.

	CIG (OR)	ECIG (OR)	Dual-users (OR)
Health literacy (ref. = limited)	0.833***	0.452***	0.632***
Sex (ref. = female)	226.384***	3.317***	76.121***
Age (ref. = ≥65)			
15–24	0.398***	0.978	4.955***
25–44	1.320***	2.222**	4.138***
45–64	1.356***	1.139	2.377***
Marital status (ref. = Married)	1.210***	1.560*	1.397*
Educational level (ref. = College or above)			
Less than junior high school	2.042***	1.313	1.184
Junior/senior high school	1.863***	1.326	1.043
Occupation (ref. = Unemployed/Others)			
Technical/Professional	0.792***	1.719	0.892
Students	0.119***	0.645	0.160***
Manual	0.917*	0.998	1.026
Commercial/Service	0.862**	1.284	1.100
Annual household income (ref. = ≥15,0000)			
0–49,999	0.864***	1.103	0.935
50,000–99,999	0.870***	0.898	0.663*
100,000–149,999	0.956	0.712	0.644**
Chronic conditions (ref. = Yes)	1.208***	1.046	1.327*

All estimates adjusted for complex sampling, gender, age, marital status, education level, Occupation, Annual household income, and Chronic conditions; outcome reference group was non-user; boldface indicates statistical significance (*p < 0.05; **p < 0.01; ***p < 0.001).

## Results

### Basic characteristics

The characteristics of the participants are shown in Table 1. A total of 60,998 qualified questionnaires were collected in the survey, of which 0.3% were ECIG-exclusive users, 19.0% were CIG-exclusive users, 0.6% were dual users, and the rest were non-tobacco users. Among the participants, 30.8% had adequate HL. Most participants were female (52.5%), middle-aged (25–64, 82.0%), married (82.1%), with junior/senior high school education (49.1%), and manual workers (51.9%). Most participants did not have chronic diseases (72.2%). The participants’ household incomes were evenly distributed (0–49,999 [30.8%], 50,000–99,999 [23.4%], 100,000–149,999 [21.2%], ≥15,0000 [24.5%]).

### Composition of ECIG use among different demographic groups

The chi-squared (χ^2^) test was performed to compare the composition of ECIG use among different demographic groups (Table 1). Statistically significant differences were observed among all groups: HL composition (χ^2^ = 161.078, *p* < 0.001), sex composition (χ^2^ = 15962.638, *p* < 0.001), age composition (χ^2^ = 602.123, *p* < 0.001), marital status composition (χ^2^ = 34.783, *p* < 0.001), educational level composition (χ^2^ = 676.157, *p* < 0.001), occupation composition (χ^2^ = 533.947, *p* < 0.001), annual household income (χ^2^ = 40.365, *p* < 0.001), and chronic condition composition (χ^2^ = 41.566, *p* < 0.001). The chi-square test results showed that the value of ps of all variables were < 0.001, indicating that these variables were correlated with ECIG use and could be included in subsequent logistic regression models as covariables.

### Factors affecting ECIG use

Multinomial logistic regression was performed to explore the factors associated with ECIG use (Table 2). In addition, there were significant associations between HL and the two patterns of ECIG use. Based on the OR values, people were more likely to use both CIG and ECIG (OR = 0.638, *p* < 0.001) than they were to exclusively use ECIG (OR = 0.457, p < 0.001). Sex was also associated with ECIG and CIG use. Males were more likely to use ECIG and CIG than females (OR_CIG_ = 226.579, p < 0.001; OR_ECIG_ = 3.352, *p* < 0.001; OR_dual use_ = 74.780, p < 0.001). Middle-aged (25–64 years) people were more likely to dual use CIG/ECIG than older (≥65) people (OR_25–44_ = 4.138, p < 0.001; OR_45–64_ = 2.377, p < 0.001). Younger people (15–24 years) were more likely to dual use CIG and ECIG (OR = 4.955, p < 0.001) than CIG-exclusive use (OR = 0.398, *p* < 0.398). ECIG-exclusive users were mainly concentrated among those aged 25–44 (OR = 2.222, *p* < 0.01). People who were single, widowed, or divorced were more likely to use ECIG and CIG (OR_CIG_ = 1.210, p < 0.001; OR_ECIG_ = 1.560, *p* < 0.05; OR_dual use_ = 1.397, p < 0.05) than married individuals. Educational level, occupation, annual household income, and chronic conditions were associated with CIG-exclusive use. However, these variables were not associated with ECIG-exclusive use. Occupation, annual household income, and chronic conditions were associated with dual use.

## Discussion

In our study, ECIG users were divided into ECIG-exclusive and CIG/ECIG dual user groups. To the best of our knowledge, this is the first study to describe the association between HL and different types of ECIG use among Chinese citizens. Our study demonstrates that HL is a protective factor against ECIG use behavior, and the effect is stronger for ECIG-exclusive use.

The HL level in this study (30.8%) was slightly higher than the national level (27.8%), but similar to that in the eastern region (31.9%) (36). This was because Zhejiang Province is located in the eastern part of China, where the urbanization rate is relatively high, and the economic level and education level are higher than the national average. Studies have shown that economic level and education level have a significant impact on residents’ HL (37, 38). The prevalence of CIG (19.7%) and ECIG (1.0%) in the study sample were both lower than the national level in 2018 (CIG 26.6%; ECIG 1.6%) (11, 30). This was mainly because the smoking rate varies greatly among different provinces, with the highest in the southwest and lowest in the eastern region (southwest China is the country’s main tobacco producing region, while eastern cities have stricter anti-smoking measures); Zhejiang Province is an eastern province with a lower smoking rate (39).

Adequate HL was significantly associated with lower odds of ECIG-exclusive use. According to previous studies, ECIG is generally considered to be less harmful than CIG but is still a health hazard (6, 40). Because HL helps people choose healthy behaviors (15, 16), people with adequate HL are more likely to be aware of the dangers of ECIG and reduce its use. However, previous studies have not reached such a conclusion (21, 23). We believe that this is mainly due to differences in the study samples. Previous studies have limited their sample groups to certain subgroups, such as college students, older adults, or those with a history of smoking. Our sample covers a wide range of people and is more representative. The variation in HL in the sample population was larger than that in previous studies; therefore, we could find an association between HL and ECIG that had not been found previously.

HL was also significantly associated with dual use after adjusting for several covariates. People with adequate HL were less likely to dual use CIG and ECIG. An American study found an association between oral HL and dual use of CIG and ECIG (21), which is consistent with our findings. As mentioned, people with high HL can realize the harm of ECIG and reduce its use. Previous studies have also shown that people with high HL will reduce the use of CIG (19); thus, people with high HL have lower odds of dual use.

We found that dual users had a higher OR than ECIG-exclusive users, indicating that people are more likely to engage in dual use of CIG/ECIG than be ECIG-exclusive users. A previous study found a high proportion of dual use among ECIG users in China (11). This is mainly because current conventional smokers are more likely to use ECIG than nonsmokers. Conventional smokers have lower HL levels than non-smokers, leaving them more receptive to ECIG products; however, conventional smokers may also have increased exposure to information about new tobacco products, such as ECIG. When conventional smokers begin using ECIG, they may abandon their CIG. However, based on the low smoking cessation rate in China (39, 41), we infer that most conventional smokers will continue to use CIG after exposure to ECIG and become dual users rather than ECIG-exclusive users.

Additionally, we found that young age was associated with ECIG use; ECIG were most popular among adolescents and young adults. This is consistent with past findings (6, 7, 11). Unsurprisingly, males were more likely to use ECIG, but the gap between males and females is smaller than that for CIG, suggesting that ECIG are more attractive to females than CIG. Because the effects of ECIG on fetal brain development are unknown (42, 43), policymakers should pay more attention to female ECIG users when developing control programs. Notably, the highest income group was the most likely to use ECIG. This implies that public knowledge about the harm of ECIG remains low. Another explanation is that ECIG were first launched in China as a luxury product targeted to high-income earners (44). Further, married individuals were less likely to use both CIG and ECIG, which may have been due to family requirements (45) or fertility concerns (46). Chronic patients are less likely to use CIG and ECIG because they curtail their unhealthy habits when they perceive that their physical condition is worsening.

### Limitations and future directions

There are some limitations in this study. First, the sample of this study was collected in Zhejiang Province. Because of differences in HL levels and smoking/vaping rates across the country, we cannot generalize these results to other provinces. Second, because this study adopted a cross-sectional research design, we cannot prove a causal relationship between HL and ECIG use. Despite the above limitations, the results of this study demonstrated an association between HL and ECIG use, providing evidence for policy makers to control EIG use. Specifically, limiting adolescents’ ECIG use can begin with HL. Future studies should continue to explore the causal relationship and specific impact mechanisms of HL and ECIG use.

## Conclusion

In conclusion, HL is a protective factor against both patterns of ECIG use, especially ECIG-exclusive use. Adequate HL was associated with lower odds of ECIG-exclusive and dual use. Thus, health policymakers and public health practitioners should consider increasing HL as a potential measure for ECIG control. Other factors associated with ECIG use were young age, male sex, unmarried status, high-income status, and absence of chronic disease. Health policymakers should pay more attention to people with these characteristics and implement targeted ECIG control measures, including improving their HL.

## Data availability statement

The raw data supporting the conclusions of this article will be made available by the authors, without undue reservation.

## Ethics statement

The studies involving humans were approved by Zhejiang Provincial Center for Disease Control and Prevention, Hangzhou, China. The studies were conducted in accordance with the local legislation and institutional requirements. Written informed consent for participation was not required from the participants or the participants' legal guardians/next of kin in accordance with the national legislation and institutional requirements.

## Author contributions

XuZ: Data curation, Investigation, Project administration, Writing – review & editing. XiZ: Writing – original draft, Writing – review & editing, Methodology, Software. SZ: Software, Writing – review & editing. LG: Methodology, Writing – review & editing. YX: Data curation, Writing – review & editing. DY: Data curation, Investigation, Writing – review & editing. XH: Validation, Writing – review & editing. ZH: Writing – review & editing. TZ: Validation, Writing – review & editing. ZW: Conceptualization, Supervision, Writing – review & editing. CC: Conceptualization, Project administration, Supervision, Writing – review & editing.
