# Supplementary material for: The association between health literacy and e-cigarette use: evidence from Zhejiang, China
Source: Front Public Health. 2024 Jan 4;11:1321457. doi: 10.3389/fpubh.2023.1321457 (PMC10794489; doi:10.3389/fpubh.2023.1321457)
Supplement: Supplementary file 1 [file Table_1.DOCX]

Supplementary Table 1 Adjusted multinomial logistic regression association between tobacco use and knowledge & attitudes

|  | CIG (OR) | ECIG (OR) | Dual-users (OR) |
| --- | --- | --- | --- |
| Knowledge and attitudes  (ref. = limited) | **0.855***** | **0.290***** | **0.616***** |
| Sex (ref. = female) | **226.222***** | **3.302***** | **75.920***** |
| Age (ref. = ≥65) |  |  |  |
| 15-24 | **0.398***** | 1.027 | **5.029***** |
| 25-44 | **1.316***** | **2.387**** | **4.175***** |
| 45-64 | **1.357***** | 1.168 | **2.394***** |
| Marital status (ref. = Married) | **1.212***** | **1.538*** | **1.396*** |
| Educational level  (ref. = College or above) |  |  |  |
| Less than junior high school | **2.055***** | 1.072 | 1.150 |
| Junior/senior high school | **1.888***** | 1.200 | 1.049 |
| Occupation  (ref. = Unemployed/Others) |  |  |  |
| Technical/Professional | **0.791***** | **1.777*** | 0.891 |
| Students | **0.118***** | 0.662 | **0.158***** |
| Manual | **0.914*** | 0.977 | 1.015 |
| Commercial/Service | **0.861**** | 1.312 | 1.101 |
| Annual household income  (ref. = ≥15,0000) |  |  |  |
| 0–49,999 | **0.866***** | 1.053 | 0.933 |
| 50,000–99,999 | **0.873***** | 0.871 | **0.665*** |
| 100,000–149,999 | 0.958 | 0.699 | **0.645**** |
| Chronic conditions (ref. = Yes) | **1.207***** | 1.049 | **1.325*** |

All estimates adjusted for complex sampling, gender, age, marital status, education level, Occupation, Annual household income, and Chronic conditions; outcome reference group was non-user; boldface indicates statistical significance (*p < 0.05; **p < 0.01; ***p < 0.001).

Supplementary Table 2 Adjusted multinomial logistic regression association between tobacco use and behavior & lifestyle

|  | CIG (OR) | ECIG (OR) | Dual-users (OR) |
| --- | --- | --- | --- |
| Behavior and lifestyle (ref. = limited) | **0.855***** | **0.420***** | **0.669**** |
| Sex (ref. = female) | **226.363***** | **3.316***** | **76.042***** |
| Age (ref. = ≥65) |  |  |  |
| 15-24 | **0.397***** | 0.998 | **4.939***** |
| 25-44 | **1.311***** | **2.230**** | **4.081***** |
| 45-64 | **1.350***** | 1.130 | **2.353***** |
| Marital status (ref. = Married) | **1.212***** | **1.571*** | **1.406**** |
| Educational level  (ref. = College or above) |  |  |  |
| Less than junior high school | **2.084***** | 1.337 | 1.235 |
| Junior/senior high school | **1.886***** | 1.331 | 1.070 |
| Occupation  (ref. = Unemployed/Others) |  |  |  |
| Technical/Professional | **0.787***** | 1.699 | 0.877 |
| Students | **0.118***** | 0.630 | **0.156***** |
| Manual | **0.916*** | 0.992 | 1.020 |
| Commercial/Service | **0.859**** | 1.283 | 1.091 |
| Annual household income  (ref. = ≥15,0000) |  |  |  |
| 0–49,999 | **0.869***** | 1.117 | 0.953 |
| 50,000–99,999 | **0.874***** | 0.908 | **0.672*** |
| 100,000–149,999 | 0.960 | 0.720 | **0.652**** |
| Chronic conditions (ref. = Yes) | **1.208***** | 1.046 | **1.328*** |

All estimates adjusted for complex sampling, gender, age, marital status, education level, Occupation, Annual household income, and Chronic conditions; outcome reference group was non-user; boldface indicates statistical significance (*p < 0.05; **p < 0.01; ***p < 0.001).

Supplementary Table 3 Adjusted multinomial logistic regression association between tobacco use and health-related skills

|  | CIG (OR) | ECIG (OR) | Dual-users (OR) |
| --- | --- | --- | --- |
| Health-related skills (ref. = limited) | **0.916**** | **0.440***** | **0.630***** |
| Sex (ref. = female) | **225.967***** | **3.327***** | **76.205***** |
| Age (ref. = ≥65) |  |  |  |
| 15-24 | **0.392***** | 0.951 | **4.869***** |
| 25-44 | **1.292***** | **2.154**** | **4.042***** |
| 45-64 | **1.348***** | 1.139 | **2.372***** |
| Marital status (ref. = Married) | **1.215***** | **1.557*** | **1.397*** |
| Educational level  (ref. = College or above) |  |  |  |
| Less than junior high school | **2.129***** | 1.394 | 1.215 |
| Junior/senior high school | **1.919***** | 1.404 | 1.071 |
| Occupation  (ref. = Unemployed/Others) |  |  |  |
| Technical/Professional | **0.784***** | 1.679 | 0.879 |
| Students | **0.117***** | 0.641 | **0.159***** |
| Manual | **0.916*** | 0.989 | 1.018 |
| Commercial/Service | **0.856**** | 1.266 | 1.087 |
| Annual household income  (ref. = ≥15,0000) |  |  |  |
| 0–49,999 | **0.873***** | 1.113 | 0.943 |
| 50,000–99,999 | **0.878**** | 0.909 | **0.669*** |
| 100,000–149,999 | 0.962 | 0.720 | **0.648**** |
| Chronic conditions (ref. = Yes) | **1.206***** | 1.047 | **1.329*** |

All estimates adjusted for complex sampling, gender, age, marital status, education level, Occupation, Annual household income, and Chronic conditions; outcome reference group was non-user; boldface indicates statistical significance (*p < 0.05; **p < 0.01; ***p < 0.001).
